# High-quality thulium iron garnet films with tunable perpendicular magnetic anisotropy by *off-axis* sputtering – correlation between magnetic properties and film strain

**DOI:** 10.1038/s41598-018-29493-5

**Published:** 2018-07-23

**Authors:** C. N. Wu, C. C. Tseng, Y. T. Fanchiang, C. K. Cheng, K. Y. Lin, S. L. Yeh, S. R. Yang, C. T. Wu, T. Liu, M. Wu, M. Hong, J. Kwo

**Affiliations:** 1 Department of Physics, National Tsing Hua University, Hsinchu, 30013 Taiwan; 2 Graduate Institute of Applied Physics and Department of Physics, National Taiwan University, Taipei, 10617 Taiwan; 3 National Nano Device Laboratories, Hsinchu, 30013 Taiwan; 4 Department of Physics, Colorado State University, Fort Collins, CO 80523 USA

## Abstract

Thulium iron garnet (TmIG) films with perpendicular magnetic anisotropy (PMA) were grown on gadolinium gallium garnet (GGG) (111) substrates by *off-axis* sputtering. High-resolution synchrotron radiation X-ray diffraction studies and spherical aberration-corrected scanning transmission electron microscope (Cs-corrected STEM) images showed the excellent crystallinity of the films and their sharp interface with GGG. Damping constant of TmIG thin film was determined to be 0.0133 by frequency-dependent ferromagnetic resonance (FMR) measurements. The saturation magnetization (M_s_) and the coercive field (H_c_) were obtained systematically as a function of the longitudinal distance (L) between the sputtering target and the substrate. A 170% enhancement of PMA field (H_⊥_) was achieved by tuning the film composition to increase the tensile strain. Moreover, current-induced magnetization switching on a Pt/TmIG structure was demonstrated with an ultra-low critical current density (j_c_) of 2.5 × 10^6^ A/cm^2^, an order of magnitude smaller than the previously reported value. We were able to tune M_s_, H_c_ and H_⊥_ to obtain an ultra-low j_c_ of switching the magnetization, showing the great potential of sputtered TmIG films for spintronics.

## Introduction

Utilizing the electron’s second fundamental characteristic—spin, advances in spintronic research such as spin-transfer torque (STT) and spin-orbit torque (SOT) have recently attracted enormous attention. Especially, the manipulation of the magnetization of a ferromagnet in using a pure spin current generated by large spin-orbit coupling materials is a fascinating topic for both fundamental research and technological application. A pure spin current injected to a ferromagnet will induce two types of SOTs, i.e., a damping-like torque (DLT) directed along $\hat{m}\times$($\hat{m}\times \hat{\sigma })$ and a field-like torque (FLT) along $\hat{m}\times \hat{\sigma }$, where $\hat{m}$ and $\hat{\sigma }$ represent the unit vectors of magnetization M and spin polarization, respectively. SOTs have been experimentally verified to perform the magnetization switching^1^, which paves the way to realize all-electrical control of the magnetization.

Among various ferromagnets, ferrimagnetic insulators (FIs) of rare earth iron garnets have attracted a great deal of interests because of their unique properties. Yttrium iron garnet (YIG)^2–5^ and lutetium iron garnet (LuIG)^6^ possess ultra-low intrinsic damping constants, which benefits spin transport studies such as long-range spin wave propagation, efficient spin pumping, SOT and spin-torque ferromagnetic resonance (ST-FMR). Moreover, thulium iron garnet (TmIG) films grown on (111)-oriented gadolinium gallium garnet (GGG) substrates were reported to show stress-induced PMA^7,8^. Recently, SOT-driven magnetization switching has been demonstrated in Pt/TmIG hetero-structures^9^. The pure spin currents injected to TmIG exert a torque and reverse the magnetization at low current densities. The pure spin currents can be induced not only by spin Hall effect (SHE) in Pt/TmIG hetero-structures, but also by Rashba-Edelstein effect (REE) in TI/TmIG hetero-structures. A charge current directed in the plane of TI induces nonequilibrium spin polarization by REE and leads to a torque acting on the magnetization of the magnetic layer^10^. A higher efficiency is expected because of the spin-momentum locking features of topological surface states. PMA can reduce the threshold switching current and maintain high thermal stability of the magnetization; the electrically insulating property of TmIG also circumvents the shunting effect. High-quality TmIG films pave the way towards ultra-low current-induced SOT for magnetization manipulation in ferromagnetic hetero-structures with high SOT efficiency, thus realizing the magnetization switching, magnetic oscillation and ultrafast chiral domain wall motion. These can be applied to data storage, magnetic random access memory, logic/memory utilizing domain wall^9^.

It was reported that TmIG films had been grown mostly by pulsed laser deposition (PLD)^7–9,11,12^. However, the small deposition area is the major shortcoming of PLD, limiting its application in industry. As an alternative way to deposit oxide thin films, we have successfully utilized *off-axis* magnetron sputtering to grow high-quality TmIG films with excellent crystallinity and PMA on GGG(111)^13^ and observed a negative magnetoresistance in TI/TmIG hetero-structures, revealing time-reversal symmetry breaking in TI^14^. The advantage of *off-axis* sputtering includes fewer ion bombardments and applications of large-scale growth. In this work, we perform the systematic study on the magnetic and structural properties of films with different Tm/Fe ratios. We have examined the excellent crystallinity of TmIG films by spherical aberration-corrected scanning transmission electron microscope (Cs-corrected STEM), and determined the damping constant of the TmIG thin films by frequency-dependent ferromagnetic resonance (FMR) measurements. Off-stoichiometric TmIG films with Fe-rich composition possess smaller lattice constants and larger *in-plane* tensile strains on GGG(111). We further demonstrated the tunability of PMA by varying the film composition, which verified that PMA in TmIG films was manipulated by the film strain, and is originated from the magnetostriction effect. Remarkably, the notable enhancement of perpendicular magnetic anisotropy field (H_⊥_) was achieved by increasing the *in-plane* tensile strain. Moreover, the current-induced magnetization switching of Pt/TmIG with the ultra-low critical current density 2.5 × 10^6^ A/cm^2^ was reported. We also estimated the real part G_r_ and the imaginary part G_i_ of spin mixing conductance in Pt/TmIG to be 1.1 × 10^15^ Ω^−1^ m^−2^ and 1.2 × 10^14^ Ω^−1^ m^−2^, respectively from the transverse resistance of Hall measurements, which implied the efficient spin transfer at the interface between Pt and TmIG. Our work of high-quality, highly tunable PMA TmIG films provides a new route to tailoring rare earth iron garnet films for spintronic application.

## Results and Discussion

### TEM and STEM images of TmIG thin film

**Figure 1:**
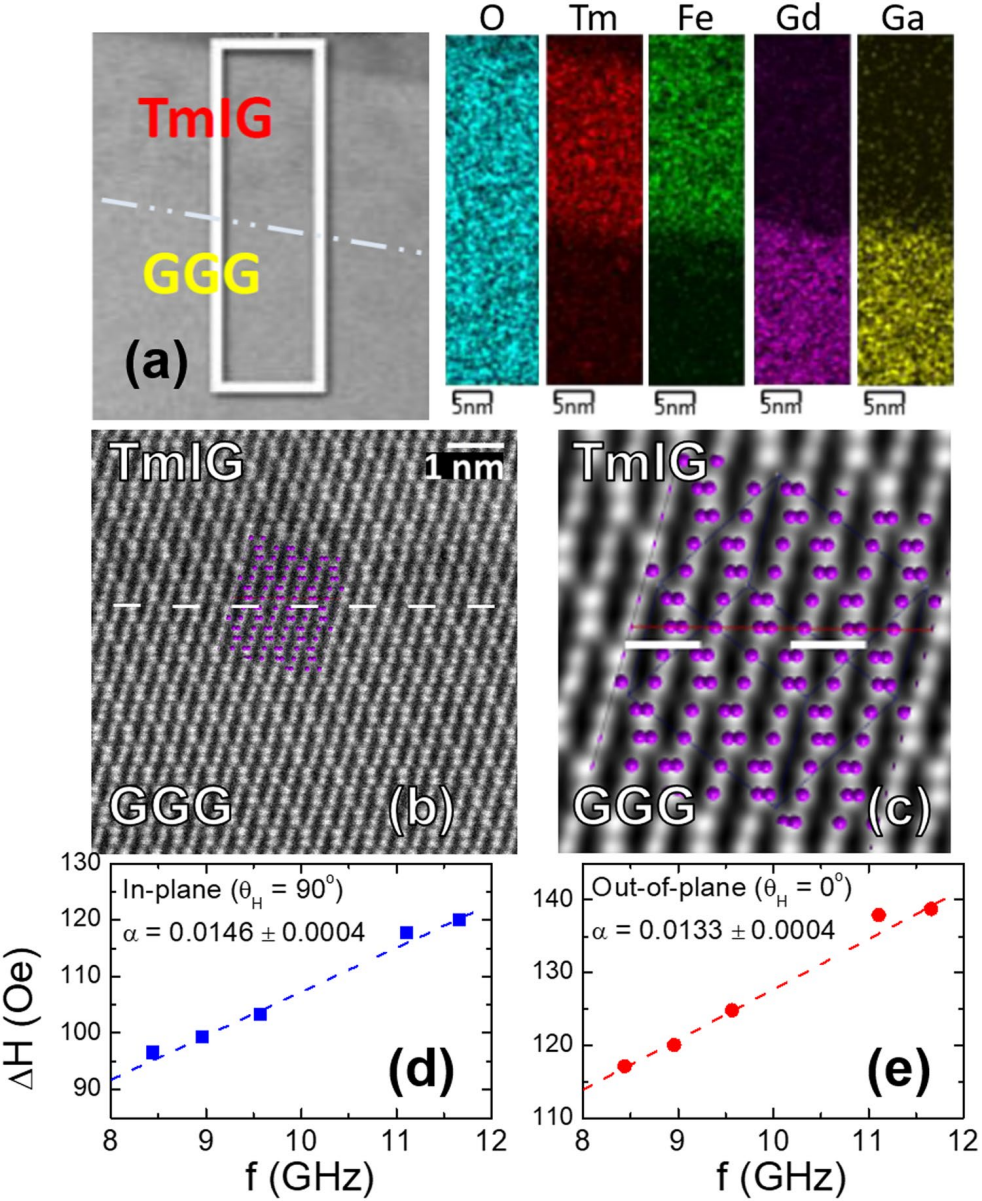
Properties of a TmIG/GGG(111) thin film grown by *off-axis* sputtering at a longitudinal distance of 7 cm. **(a)** The TEM image and the EDS element mapping images of O, Tm, Fe, Gd and Ga elements colored in blue, red, green, purple and yellow, respectively. **(b)** Cs-corrected STEM HAADF images with zone axis: [−1, −2, 3]. **(c)** A magnified and noise filtered image of **(b)**. The purple dots denote the locations of Gd in GGG and Tm in TmIG, respectively, plotted by a crystallographic program. The white dash lines denote the interface of TmIG/GGG. **(d,e)** The FMR data measured with a static magnetic field applied in the plane, and perpendicular to the TmIG film, respectively.

Figure 1 shows the structural and magnetic properties of the optimized *off-axis* sputtered TmIG thin film on GGG(111) with a longitudinal distance of 7 cm^13^. The cross-sectional TEM image and the energy-dispersive X-ray spectroscopy (EDS) element mapping images shown in Fig. 1(a) show the uniform composition distribution in each layer and show no obvious chemical intermixing at the interface of the TmIG/GGG thin film. Figure 1(b,c) are the cross-sectional high-angle annular dark-field (HAADF) images of Cs-corrected STEM with atomic resolution, showing excellent crystallinity of the TmIG. The spots with brighter contrast denote the heavier atoms in the samples, and the spots match very well with the purple dots representing heavier atoms of Gd in GGG and Tm in TmIG thin films plotted by a crystallographic program. Furthermore, a sharp and nearly perfect interface between the TmIG and GGG layers was observed, showing no visible interfacial defect and dislocation. The STEM results show that the TmIG film was epitaxially grown on the GGG substrate with exactly the same crystal structure and the same orientation with the sharp interface. The STEM results are in agreements with the XRD results showing the extended Pendellosung fringes.

### Damping constant of TmIG thin film

We conducted the frequency-dependent ferromagnetic resonance (FMR) measurements on the TmIG films to extract the damping constant. The linewidth of the FMR spectrum (ΔH) increased linearly as a function of the microwave frequency with the magnetic field applied out of plane, and in the plane as in Fig. 1(d,e), respectively. The damping constants α was extracted by fitting ΔH as a function of frequencies (f = ω/2π),

1 $${\rm{\Delta }}{\rm{H}}=\frac{2\alpha }{\sqrt{3}|\gamma |}\frac{\omega }{2\pi }+{\rm{\Delta }}{H}_{0}$$

where γ denotes the gyromagnetic ratio and ΔH_0_ denotes the film inhomogeneity line broadening. The damping constant α of TmIG film was then determined to be 0.0133 with the field out of plane, and 0.0146 with the field in the plane. The slightly larger α value measured with the magnetic field applied in the plane than the out-of-plane might be an indication of the two-magnon scattering (TMS)^15–17^. The TMS is due to the film inhomogeneity (such as grain boundaries and voids) and makes significant contributions when the applied magnetic field is not along the film normal direction. The contribution of TMS to α is minimized when the field is normal to the sample plane when conducting the FMR measurements.

### TmIG film composition tuning: tensile strain and magnetic properties

**Figure 2:**
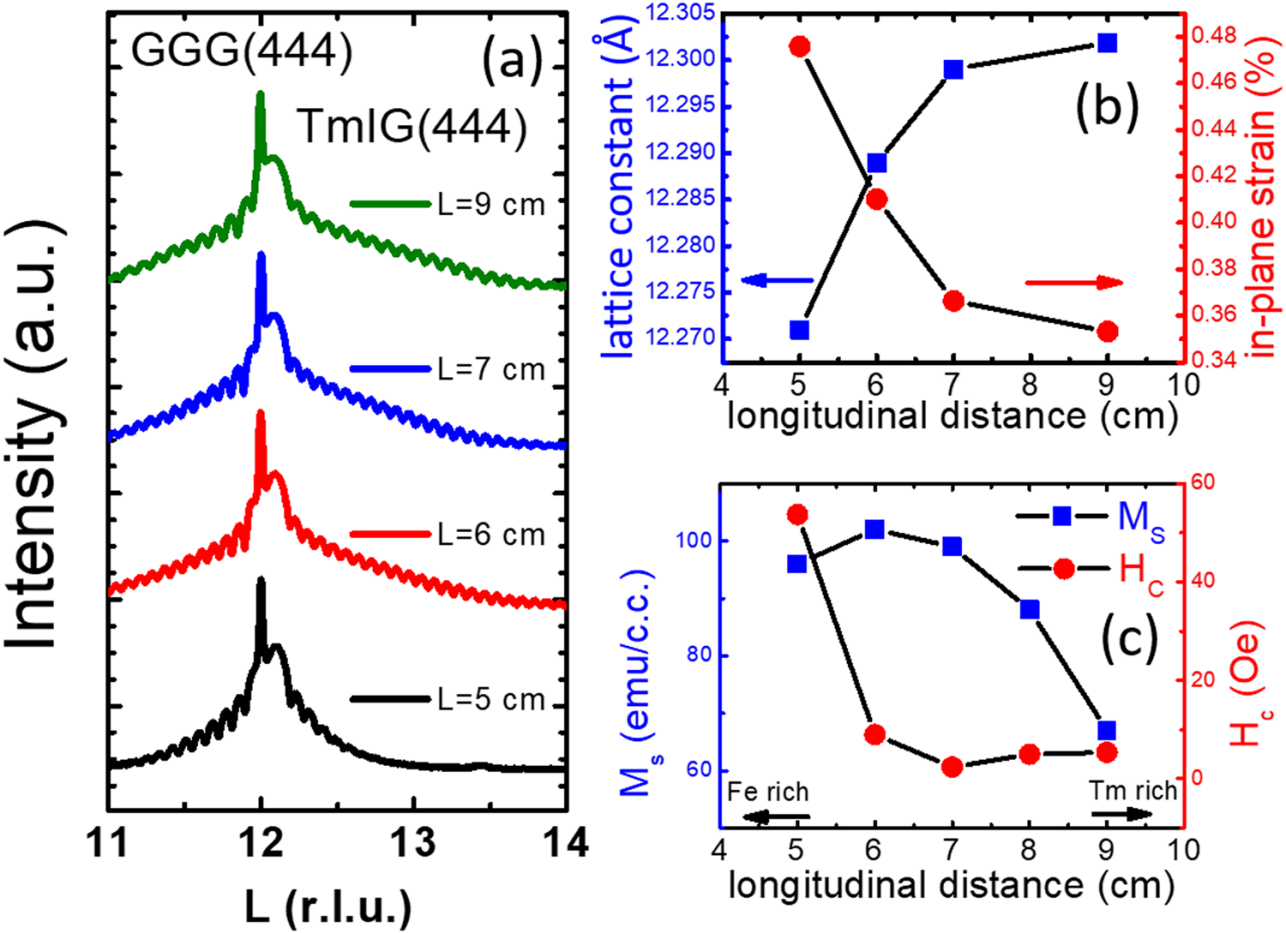
**(a)** XRD L-scan results for TmIG films grown at various longitudinal distances. **(b)** Out-of-plane lattice parameters and the calculated strain as a function of the longitudinal distance. **(c)** M_s_ and H_c_ as a function of the longitudinal distance.

The surface normal XRD scans of the samples A, B, C, D and E are shown in Fig. 2(a). Note that these five TmIG films were sputtered at longitudinal distance L of 5, 6, 7, 8, and 9 cm, respectively, varying the composition and the strain in the films. Clear Pendellosung fringes in all TmIG films exhibited the excellent film quality. Out-of-plane lattice constants of samples A, B, C and E were 12.274 Å, 12.289 Å, 12.299 Å and 12.302 Å, respectively. The gradual increase of the out-of-plane lattice constant reflected a Tm-richer composition as L increased, consistent with the previous composition investigation^13^. The Tm: Fe ratio of samples are determined by Rutherford backscattering spectrometry (RBS) and X-ray photoelectron spectroscopy (XPS) in the Supplementary information S1. For a reference TmIG sample grown at L = 7 cm, the Tm: Fe ratio was measured as 0.57 by RBS. The Tm: Fe ratios are determined as 0.43 ± 0.03, 0.51 ± 0.05, 0.59 ± 0.05, and 0.62 ± 0.05 for samples grown at L = 5, 6, 8, and 9 cm, respectively, by XPS. A possible mechanism for the dependence of stoichiometry on longitudinal distance L is the difference in the scattering of Fe (atomic mass = 55.84 amu) and Tm (168.93 amu) atoms by Ar (39.95 amu) and O_2_ (31.99 amu) ambiance, resulting in the different spatial distribution of sputtered Fe and Tm atoms. To calculate the in-plane film strain ε∥, we used the elastic deformation model^8^. We calculated and summarized the out-of-plane lattice constants and the in-plane strain in Fig. 2(b). We show that the in-plane tensile strain can be tuned systematically from 0.353% to 0.476% with decreasing L by *off-axis* sputtering without altering the substrates.

### Enhancement of perpendicular magnetic anisotropy field by tensile strain

The 5 samples A-E all displayed PMA, as shown by MH loops measured by AGM. The magnetization (M_s_) and the coercive field (H_c_) were characterized and summarized in Fig. 2(c). The samples B and C had the room temperature maximum M_s_ of 99–102 emu/cm^3^ with a minimal H_c_ of 2.4 Oe. To clarify how the off-stoichiometry affects the magnetic properties, we refer to discussions below. The moment of rare earth iron garnet sublattice can be calculated by calculating the total moment of 3 iron ions on the tetrahedral sites minus the moment of 2 iron ions on the octahedral sites minus the moment of 3 rare earth ions (Σ m = Σ m_Fe tet_ − Σ m_Fe oct_ − Σ m_rare earth_). In the Fe-rich films, the Fe would occupy the Tm site and reduce the total moment, because of the larger moment of Fe than that of Tm^18^. As for the Tm-rich films, we observed the moment decreasing. The tetrahedral sites are much smaller than the octahedral sites for the Tm to occupy. However, if Tm ions occupied the octahedral sites, the total moment would increase. Based on our results, the magnetization seems not to be dominated by the occupation of Tm ions on the octahedral sites. On the other hand, in Tm-rich films, there is another possibility that Fe vacancies appear^19^. The Fe vacancies on the tetrahedral sites reduce the magnetic moments while Fe vacancies on the octahedral sites increase the magnetic moments. They further reported that the Fe vacancies on the tetrahedral sites remained relatively constant than that on octahedral sites, which implied that the Fe vacancies on the tetrahedral sites may dominate in slightly off-stoichiometric iron garnets. Our Tm-rich film is only off-stoichiometric to a smaller amount than their reported iron garnets. Therefore, we infer that the reduction of the magnetic moment in our Tm-rich films could be the results of domination of Fe vacancies on the tetrahedral sites. For H_c_, pinning of domain walls increases the H_c_; nucleation process decreases the H_c_. The H_c_ above 2.4 Oe might be due to the pinning of domain walls, possibly induced by the point defects such as element substitutions and vacancies often present in off-stoichiometric films^20–23^. TmIG films with low H_c_ require less current-induced effective field to switch the magnetization. We have measured the reduced current density of magnetization switching. The low H_c_ indicates higher magnetization switching efficiency although it may have less thermal stability. However, our ability to tune the H_c_ from 2.4 Oe to 54 Oe by *off-axis* sputtering offer a clear advantage when balancing between thermal stability and magnetization switching efficiency.

**Figure 3:**
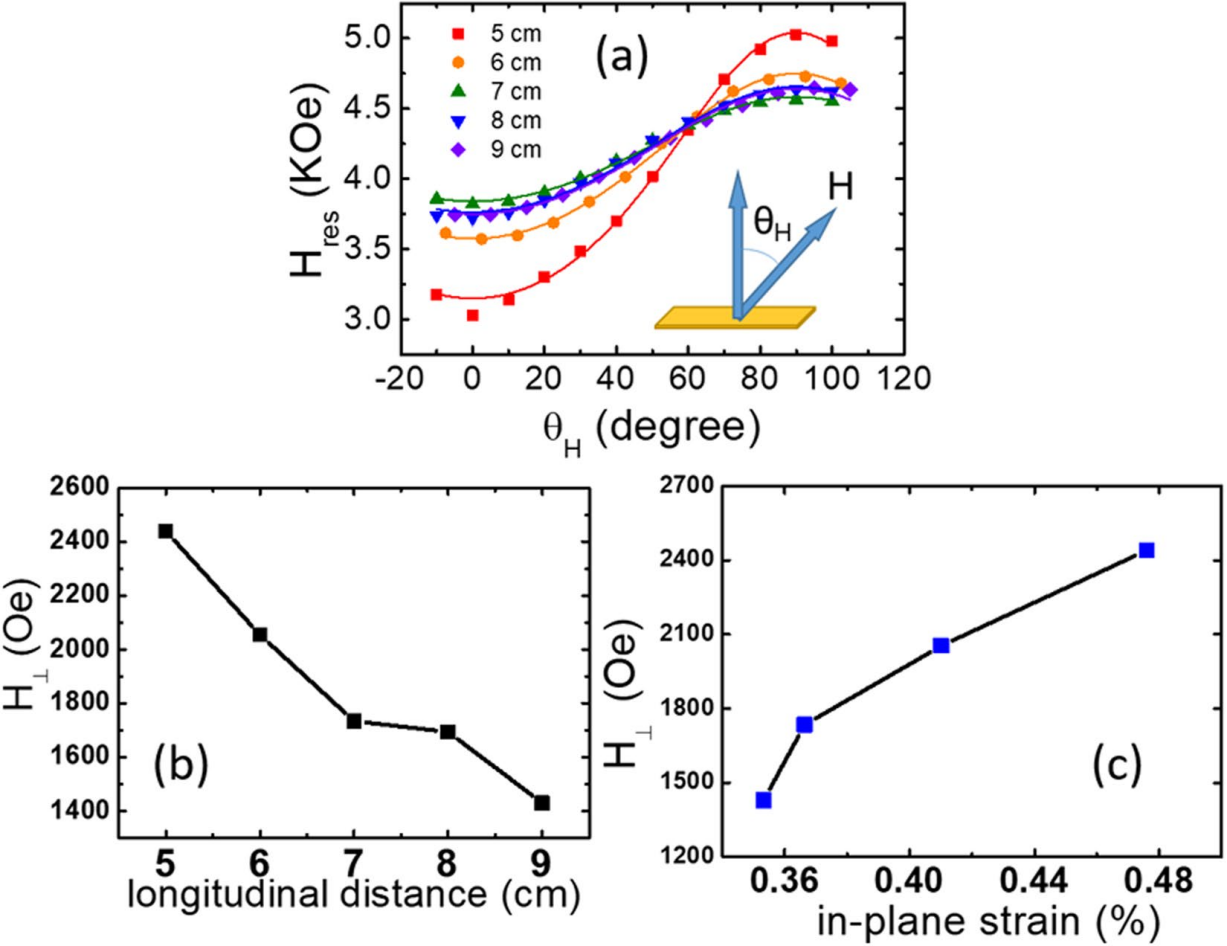
**(a)** The summary of angle-dependent FMR results. The dots denote the experimental data, and the lines denote the fitting results. θ_H_ is the angle of magnetic field with respect to the film normal. **(b)** Fitted H_⊥_ as a function of longitudinal distance. **(c)** Fitted H_⊥_ as a function of in-plane strain.

Figure 3(a) shows the FMR results. The resonance fields (H_res_) of all the samples were summarized as a function of θ_H_. The out-of-plane resonance fields of the samples are always smaller than the in-plane resonance fields, showing the PMA in the TmIG films. Figure 3(a) shows the well-fitted experimental data from angle-dependent FMR measurements, exhibiting the gradually varying 4πM_eff_ with respect to samples of different L. The effective magnetization 4πM_eff_ was determined by fitting the data in Fig. 3(a). The H_⊥_ was extracted from M_s_ and 4πM_eff_, according to 4πM_eff_ = 4πM_s_ − H_⊥_^24^. Figure 3(b) shows the calculated H_⊥_ of samples A to E by fitting the measured data in Fig. 3(a,c) displays the tensile strain dependence of H_⊥_ for samples with different Tm: Fe ratios. The H_⊥_ was enhanced significantly from 1429 Oe to 2439 Oe (170%). The results have revealed the enhancement of H_⊥_ with larger tensile strain by tuning the film composition, in excellent agreement with the results obtained by changing the substrate lattice constant^7,8,11^. The ability to tune M_s_, H_c_ and H_⊥_ of TmIG by *off-axis* sputtering showed a great potential for ultralow-dissipation spintronic devices based on FIs.

### Current-induced magnetization switching

**Figure 4:**
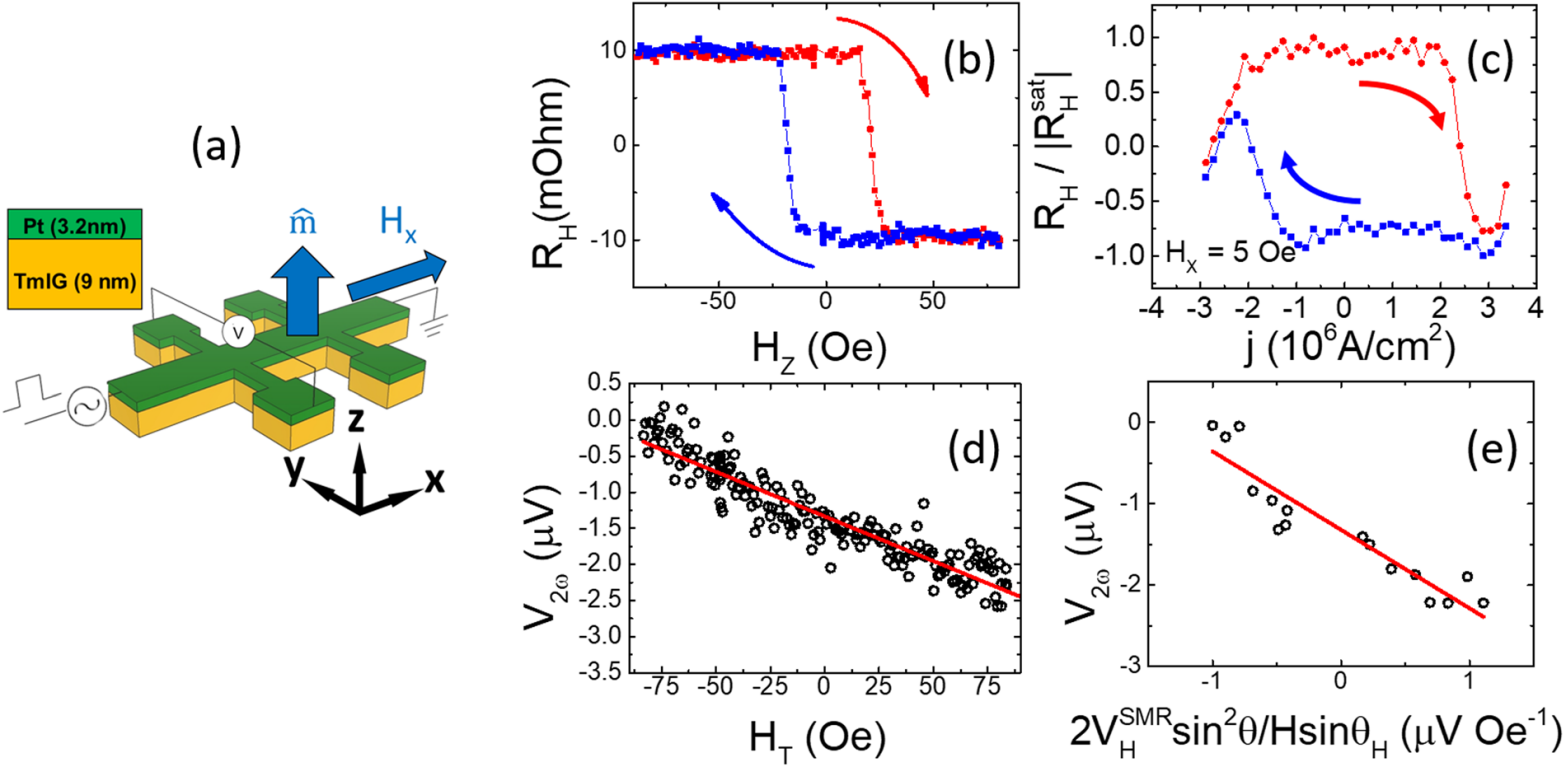
**(a)** Illustrations of Pt/TmIG bilayer structure made into a Hall bar device with coordinate systems and the electrical measurement set-up. **(b)** Hall resistance R_H_ measured as a function of out-of-plane field H_z_ (a constant offset is subtracted). **(c)** The switching loop of the Hall bar as a function of the injected current density j for a given field of H_x_ = 5 Oe. The critical current j_c_ is ~2.5 × 10^6^ A/cm^2^. **(d)** The second Hall voltage measured as a function of in-plane field H_T_ along y direction with an applied current density of j_r.m.s._ = 1.88 × 10^6^ A/cm^2^. **(e)** V_2ω_ plotted as a function of $(2{{\rm{V}}}_{{\rm{H}}}^{{\rm{SMR}}}{\sin }^{2}\,{\rm{\theta }})/({\text{Hsin}{\rm{\theta }}}_{{\rm{H}}})\,$to calculate the H_DL_ by fitting the linear slope [red line in **(e)**].

For the current-induced magnetization switching experiment, a Pt/TmIG bilayer was patterned into a Hall bar for the Hall effect measurements, as shown in Fig. 4(a). The Hall resistance as a function of the out-of-plane magnetic field H_Z_ in Fig. 4(b) shows that the magnetization of TmIG switched by the out-of-plane magnetic field. The measured Hall resistance R_H_ after applying the electric pulses are plotted as a function of the injected current density (j) with the low in-plane field H_X_ = 5 Oe, as shown in Fig. 4(c). The magnetization was switched with an ultralow critical current density j_c_ ~ 2.5 × 10^6^ A/cm^2^, one order of magnitude smaller than the first reported value (j_c_ ~ 1.8 × 10^7^ A/cm^2^) with an in-plane field H_X_ = 500 Oe in Pt/TmIG by Avci *et al*.^9^. Our result was also comparable to the switching current density (j_c_ ~ 6.0 × 10^6^ A/cm^2^) with a >35 Oe in-plane field later reported by Avci *et al*.^20^. In order to explain our low critical current density, we adopted the spin Hall magnetoresistance theory^25^ to characterize the spin transfer at the interface between Pt and TmIG by examining the spin mixing conductance, and presented the calculation in details in Supplementary Information S2. Assuming the spin Hall angle and the spin diffusion length of Pt to be 0.08^26^ and 1.4 nm^25^ respectively, we estimated the real part G_r_ and the imaginary part G_i_ of the spin mixing conductance to be 1.1 × 10^15^ Ω^−1^ m^−2^ and 1.2 × 10^14^ Ω^−1^ m^−2^, respectively, larger than the previously reported values in Pt/TmIG^9,27^. This may support the more efficient spin transmission at the interface and lead to the smaller current for the switching. The effective torque field was measured by harmonic Hall measurements. The second-harmonic Hall voltages (V_2ω_) were measured and plotted as a function of the in-plane transverse field (H_T_), as shown in Fig. 4(d). The V_2ω_ is given by the formula^9,28^.

2 $${{\rm{V}}}_{2{\rm{\omega }}}=(2{{\rm{V}}}_{{\rm{H}}}^{{\rm{SMR}}}\,{\sin }^{2}{\rm{\theta }})\frac{{{\rm{H}}}_{{\rm{DL}}}}{{{\rm{H}}}_{{\rm{T}}}\,\sin \,{{\rm{\theta }}}_{{\rm{H}}}}$$

when the H_T_ tilts the sample magnetization in the y-z plane, where H_DL_ is the effective field associated with damping-like SOT. θ and θ_H_ present the angle of the magnetization and applied field to the z-axis, respectively. ${{\rm{V}}}_{{\rm{H}}}^{{\rm{SMR}}}$ is the transverse manifestation of the Hall voltage induced by the spin Hall magnetoresistance. In order to calculate the H_DL_, we plotted the V_2ω_ as a function of $(2{{\rm{V}}}_{{\rm{H}}}^{{\rm{SMR}}}{\sin }^{2}\,{\rm{\theta }})/({{\rm{H}}}_{{\rm{T}}}{\sin {\rm{\theta }}}_{{\rm{H}}})$, and H_DL_ was then determined by fitting the data and calculating the slope. We obtained H_DL_ = 0.97 ± 0.1 Oe per j_r.m.s._ = 1.88 × 10^6^ A/cm^2^; namely: H_DL_ = 10.9 Oe per j_r.m.s._ = 2.1 × 10^7^ A/cm^2^, which is comparable to the reported value (H_DL_ = 12.3 Oe per j_r.m.s._ = 2.1 × 10^7^ A/cm^2^)^9^.

## Conclusion

We have grown high-quality TmIG films with PMA on GGG(111) by *off-axis* sputtering. Excellent crystallinity of TmIG films was observed from Cs-corrected STEM images with a sharp interface and synchrotron radiation XRD. Damping constant of the TmIG thin film was measured to be 0.0133. We have demonstrated the enhancement of H_⊥_ from 1429 Oe to 2439 Oe (170%) by adjusting the TmIG film composition to be Fe rich, thereby increasing the tensile strain. Moreover, we also have demonstrated current induced switching with a very low j_c_ of 2.5 × 10^6^ A/cm^2^ and determined the effective torque field H_DL_ = 0.97 ± 0.1 Oe per j_r.m.s._ = 1.88 × 10^6^ A/cm^2^. Spin mixing conductance G_r_ and G_i_ were estimated to be 1.1 × 10^15^ Ω^−1^ m^−2^ and 1.2 × 10^14^ Ω^−1^ m^−2^ respectively. Our ability to tune M_s_, H_c_ and H_⊥_ in *off-axis* sputtered TmIG films and the demonstration of current-induced magnetization switching hold excellent potential for spintronic applications.

## Methods

### Sample preparation

The TmIG films were grown on GGG(111) substrates by *off-axis* RF sputtering and the detailed sputtered parameters were given in ref.^13^. We have prepared five TmIG films as samples A, B, C, D, and E with respective longitudinal distance L (between the target and the substrates) of 5, 6, 7, 8, and 9 cm in order to vary the film Tm: Fe compositional ratio and tune the strain to achieve the best magnetic properties. The O_2_/Ar ambient pressure was at 3.5 mtorr and the RF power was at 40 Watt.

### Structural characterization

The cross-sectional samples for TEM were prepared by conventional mechanical polishing. The element mappings were conducted by EDS. The cross-sectional HAADF images of TmIG thin films were obtained by Cs-corrected STEM to show the crystallinity and the interface of TmIG/GGG. The STEM investigations were conducted on a JEOL-2100F microscope with a CEOS spherical-aberration corrector, so that we can obtain the high-quality STEM images with an atomic resolution. The HAADF images taken from very high angle are highly sensitive to the variations in the atomic number of atoms in the sample. XRD using synchrotron radiation has been employed to study crystallography and the strain of the sputtered TmIG films epitaxially grown on GGG.

### Magnetic characterization

The magnetic properties, such as the M_s_, the H_c_ were measured using AGM. The damping constants α and H_⊥_ were measured using a series of X-band TE_102_ mode microwave cavities.

### Current-induced magnetization switching measurements

To conduct the current-induced magnetization switching experiments^9^, an e-beam evaporated Pt thin film 3.2 nm thick was grown on a TmIG thin film 9 nm thick. The sample was patterned into a Hall bar with dimension 800 μm × 100 μm for Hall effect measurements. An in-plane magnetic field H_X_ of 5 Oe was applied along the direction of applied current. Electric pulses with 5 milliseconds duration were applied by Keithley 2400. Second-harmonic measurements using SR830 lock-in amplifier were performed with a frequency of 500 Hz and an applied current density of 1.88 × 10^6^ A/cm^2^.

### Data availability statement

The datasets generated during and/or analyzed during the current study are available from the corresponding author on reasonable request.

## Electronic supplementary material

Supplementary Information
