# Supplementary material for: High-quality thulium iron garnet films with tunable perpendicular magnetic anisotropy by off-axis sputtering – correlation between magnetic properties and film strain
Source: Sci Rep. 2018 Jul 23;8:11087. doi: 10.1038/s41598-018-29493-5 (PMC6056423; doi:10.1038/s41598-018-29493-5)
Supplement: Supplementary file 1 — Supplementary Information [file 41598_2018_29493_MOESM1_ESM.docx]

**High-quality thulium iron garnet films with tunable perpendicular magnetic anisotropy by *off-axis* sputtering – correlation between magnetic properties and film strain**

**C. N. Wu^1^, C. C. Tseng^1^, Y. T. Fanchiang^2^, C. K. Cheng^2^, K. Y. Lin^2^, S. L. Yeh^1^, S. R. Yang^1^, C. T. Wu^3^, T. Liu^4^, M. Wu^4^, M. Hong^2*^ and J. Kwo^1*^**

^1^Department of Physics, National Tsing Hua University, Hsinchu 30013, Taiwan. ^2^Graduate Institute of Applied Physics and Department of Physics, National Taiwan University, Taipei 10617, Taiwan. ^3^National Nano Device Laboratories, Hsinchu 30013, Taiwan. ^4^Department of Physics, Colorado State University, Fort Collins CO 80523, USA. Correspondence and requests for materials should be addressed to J.K. (email: [raynien@phys.nthu.edu.tw](mailto:raynien@phys.nthu.edu.tw)) and M.H. (email: [mhong@phys.ntu.edu.tw](mailto:mhong@phys.ntu.edu.tw))

**S1. Tm: Fe ratio measurement by XPS and RBS**

We have conducted XPS measurements to determine the Tm: Fe ratios of samples grown with different longitudinal distances (L) by using the method reported in our previous work^1^. In order to prevent charging effect, TmIG films were grown on Si substrates for XPS measurements. The Fe 2p and Tm 4d core-level spectra were measured as shown in Fig. S1-1. The area of each spectrum was determined by calculating the area between the spectrum and the Shirley background. For the reference sample, we measured the Tm: Fe ratio of a TmIG sample grown on GGG(111) at L = 7 cm and with a thicker TmIG layer (110 nm) by Rutherford backscattering spectrometry (RBS). (RBS was conducted by Ms. Mao Wang and Dr. Shengqiang Zhou, Helmholtz-Zentrum Dresden-Rossendorf, Institute of Ion Beam Physics and Materials Research, Bautzner Landstr. 400, 01328, Dresden, Germany.) The Tm: Fe ratio of this sample was determined as 0.57 by fitting the RBS results, as shown in Fig. S1-2. Then, we used this value as a reference to determine the Tm: Fe ratios of the other samples grown with alternative L by calculating the areal ratios of Fe 2p and Tm 4d spectra and comparing to that of the reference sample. The Tm: Fe ratios are then determined as 0.43±0.03, 0.51±0.05, 0.59±0.05, and 0.62±0.05 for samples at L = 5, 6, 8, and 9 cm, respectively.


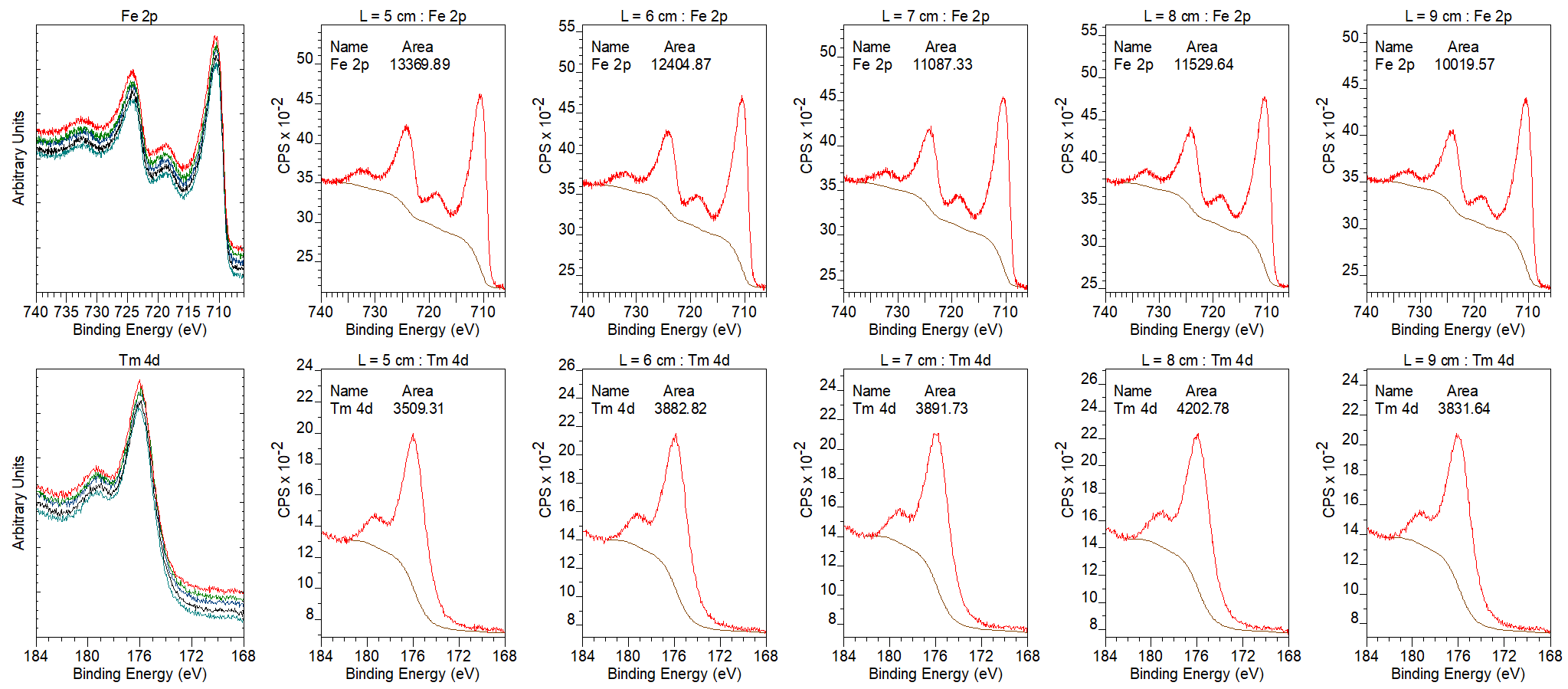


Figure S1-1 XPS spectra of the samples grown with different longitudinal distances (L). The red lines are the XPS results, and the black lines denote the Shirley backgrounds.


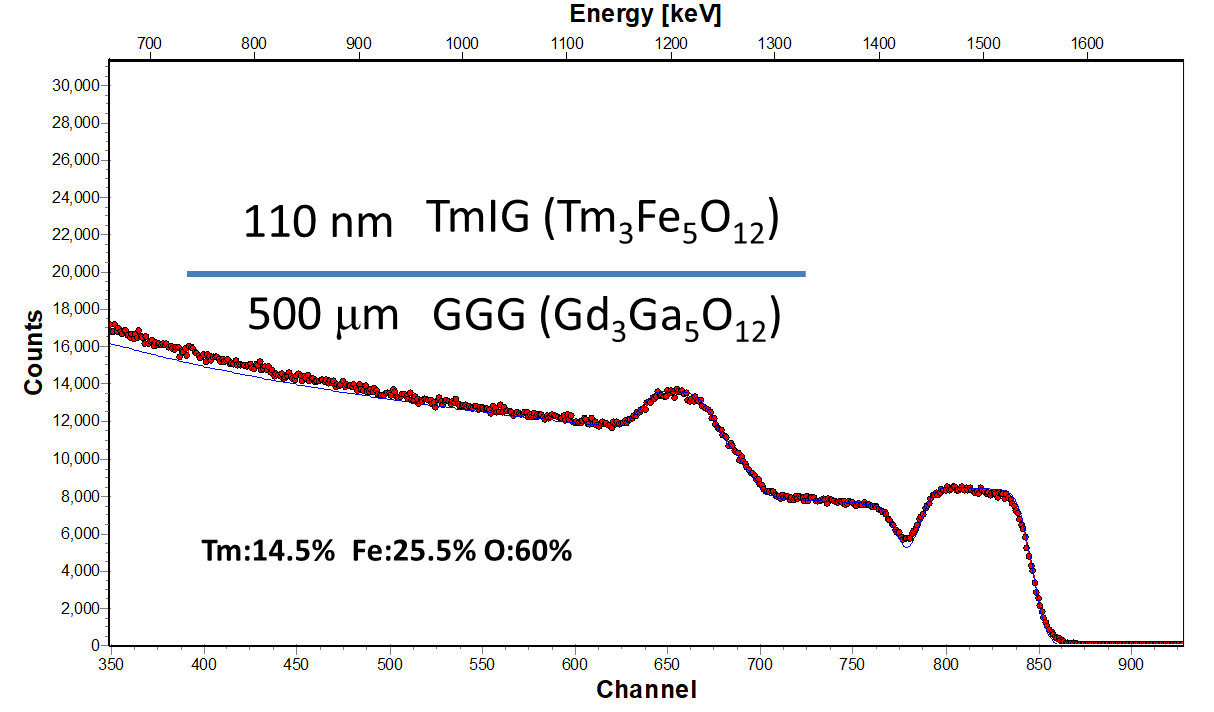


Figure S1-2 The Rutherford backscattering spectrometry (RBS) results (red dots) and the simulation (blue line) of a sample grown at L = 7 cm and 110 nm thick TmIG layer.

**S2. Estimation of spin mixing conductance G**

In order to characterize the spin transfer at the interface between Pt and TmIG, we adopted spin Hall magnetoresistance (SMR) theory to calculate the G^2^. According to the SMR model, the transverse resistivity ρ_trans_ in Pt/TmIG can be expressed as

ρ_trans_ = Δρ_1_m_x_m_y_ + Δρ_2_m_z_ (S-1)

where m_i_ denotes the i-component of the unit magnetization of TmIG. Δρ_1_ and Δρ_2_ have the following relations:

$\frac{{\Delta\rho}_{1}}{\rho}$ $\approx$ ${\theta_{SH}}^{2}\frac{\lambda}{d_{N}}\frac{2\lambda G_{r}{tanh}^{2}\frac{d_{N}}{2\lambda}}{\sigma+2\lambda G_{r}coth\frac{d_{N}}{\lambda}}$ (S-2)

$\frac{{\Delta\rho}_{2}}{\rho}$ $\approx$ ${\theta_{SH}}^{2}\frac{\lambda}{d_{N}}\frac{2\lambda{\sigma G}_{i}{tanh}^{2}\frac{d_{N}}{2\lambda}}{{(\sigma+2\lambda G_{r}coth\frac{d_{N}}{\lambda})}^{2}}$ (S-3)

where ρ, $\sigma$, d_N_, $\lambda$, $\theta$_SH_, G_r_ and G_i_ represent Pt longitudinal resistivity, conductivity, film thickness, spin diffusion length, spin Hall angle, the real part of G and the imaginary part of G respectively. ρ of our 3.2 nm Pt was measured to be 75.2 μΩ-cm. From the angle-dependent electrical measurement in Fig. S2, we extracted the Δρ_1_ of 9.7×10^-2^ μΩ-cm; we also obtained Δρ_2_ of 3.07×10^-3^ μΩ-cm from anomalous Hall measurement in Fig. 4(b). Assuming the spin Hall angle and the spin diffusion length of Pt to be 0.08 and 1.4 nm respectively, we estimated G_r_ to be 1.1×10^15^ Ω^-1^m^-2^ and G_i_ to be 1.2×10^14^ Ω^-1^m^-2^.

^
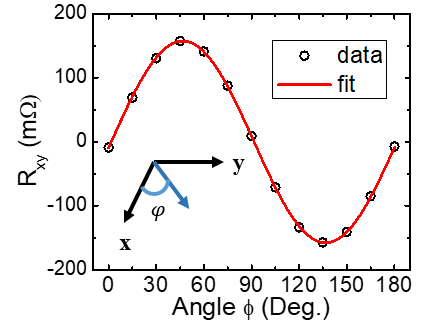
^

Figure S2 Summary of transverse resistances as a function of the direction which the magnetic field was applied along. A fit to the data according to equation (S-1) reveals Δρ_1_ of 9.7×10^-2^ μΩ-cm. $\varphi$ is defined in the inset. X-axis and y-axis lie in the film plane. Current was applied along x-axis and voltage was measured along y-axis.

**Reference**

1. C. N. Wu et al., AIP Advances **8**, 055904 (2018).

2. Y. T. Chen et al., Phys. Rev. B **87**, 144411 (2013).
